# Natural sea water and artificial sea water are not equivalent in plastic leachate contamination studies

**DOI:** 10.12688/openreseurope.17112.3

**Published:** 2024-10-17

**Authors:** Clemens Vinzenz Ullmann, Maria Ina Arnone, Eva Jimenez-Guri

**Affiliations:** 1 Department for Earth and Environmental Sciences, Faculty of Environment, Science and Economy, University of Exeter, Penryn, England, UK; 2 Biology and Evolution of Marine Organisms, Stazione Zoologica Anton Dohrn Napoli, Naples, Campania, Italy; 3 Centre for Ecology and Conservation, University of Exeter, Penryn, England, UK

**Keywords:** Plastic leachates, Zinc, Natural sea water, Artificial sea water, Development, Ciona intestinalis

## Abstract

**Background:**

Plastic contamination is one of the concerns of our age. With more than 150 million tons of plastic floating in the oceans, and a further 8 million tons arriving to the water each year, in recent times the scientific community has been studying the effects these plastics have on sea life both in the field and with experimental approaches. Laboratory based studies have been using both natural sea water and artificial sea water for testing various aspects of plastic contamination, including the study of chemicals leached from the plastic particles to the water. We set out to test this equivalence, looking at the leaching of heavy metals form plastic particles.

**Methods:**

We obtained leachates of polyvinyl chloride plastic pre-production nurdles both in natural and artificial sea water and determined the elements in excess from untreated water by Inductively coupled plasma – optical emission spectrometry. We then used these different leachates to assess developmental success in the tunicate *Ciona intestinalis* by treating fertilised eggs through their development to hatched larvae.

**Results:**

Here we report that chemical analysis of polyvinyl chloride plastic pre-production pellet leachates shows a different composition in natural and artificial sea water. We find that the zinc leaching from the plastic particles is up to five times higher in natural seawater than in artificial seawater, and this can have an effect in the toxicological studies derived. Indeed, we observe different effects in the development of *C. intestinalis* when using leachates in natural or artificial sea water. We also observe that not all artificial sea waters are suitable for studying the development of the tunicate *C. intestinalis*.

**Conclusions:**

Our results show that, at least in this case, both types of water are not equivalent to produce plastic leachaetes and suggest that precaution should be taken when conclusions are derived from results obtained in artificial sea water.

## Introduction

Plastic pollution is ubiquitous and persistent. In marine ecosystems, it has emerged as a significant concern: large plastic items can entangle marine animals, cause physical injuries, and alter or disrupt habitats; smaller plastic items can be ingested by organisms, and even be transferred to internal organs and cells ^1, 2^. Plastics also can contain chemical additives which are added to the polymers during the manufacturing process to enhance desired characteristics, such as plasticizers, biocides, flame retardants, foaming agents, pigments, stabilisers, and many more (summarised in a comprehensive review in ^3^), or that can be adsorbed and accumulated from the environment ^4, 5^. These pollutants can then be transferred from the plastic into the aquatic systems ^4– 10^. Multiple reports have studied the effect of plastic leachates in several aquatic organisms. Laboratory experiments have shown that plastic leachates increase oxidative stress ^10– 12^ and impair embryo and larval development ^6, 7, 9, 10, 13– 17^.

These studies used leachates from different types of plastic items: farming gear, pre-production plastic pellets, plastic toys, plastic bags, as well as virgin plastics and others. The protocols for lixiviation of these different studies vary slightly, and recently a study has been published to provide a unified protocol for further studies ^18^. Variations include lixiviation time, rotation speed of the samples during lixiviation, and plastic concentrations. However, the source of seawater used to obtain the leachates, provided it is not contaminated, has not been suggested as one of the variables to take into account, as both artificial seawater (ASW) and natural filtered seawater (FSW) have been accepted as equivalent ecologically relevant media for ecotoxicity tests with marine organisms ^18, 19^. As such, different studies use either ASW ^6, 8, 16, 20^ or FSW ^9, 10, 12, 14, 15, 17^ to obtain their leachates.

We have previously used pre-production polyvinyl chloride (PVC) pellet leachates in toxicology studies in sea urchin adults and embryos ^9, 10, 12^ and chemical analysis of the leachates by inductively coupled plasma – optical emission spectrometry (ICP-OES) has shown that they leach high amounts of zinc into natural seawater. These leachates inflicted very important developmental abnormalities in a large number of invertebrate taxa larvae ^9, 10, 21^. However, we wanted to know if the composition of the leachates would be similar when using artificial sea water, to test whether ASW and FSW are equivalent in laboratory tests. Here we obtain leachates of pre-production PVC pellets in three types of ASW as well as in FSW and in double deionised water (ddW) to compare their elemental content, as well as their effect on developing embryos of the tunicate *Ciona intestinalis* to confirm if they are equivalent media for obtaining heavy metal leachates of plastic particles and to perform ecotoxicology studies. In this study, we did not test for the leaching of other molecules, including organic molecules, which are known to leach from plastic pellets ^4, 5^.

## Methods

### PVC plastic leachate preparation

Commercial PVC plastic pre-production nurdles were purchased from Northern Polymers and Plastics Ltd. (UK). These were flexible tubular transparent pellets with a slight blue hue and average dimensions of 3.2 × 3.7 mm. Leachates from these plastic pellets were obtained as described in Rendell-Bhatti *et al*., 2020 with small modifications. In brief, PVC plastic pellets were added to natural seawater, artificial seawater or distilled water at a concentration of 6.5% (w/v), equivalent to 10% (v/v). Pellets were left to leach in each type of water in a glass bottle on a platform shaker at 60 rpm at 18°C in the dark for 72 h. Leachates were obtained by filtering the treated waters through filter paper in order to remove particles. Working solutions were obtained by diluting the leachates to the desired concentration with the relevant type of water.

Natural sea water was obtained from Falmouth bay, UK, and filtered using a 0.22 µm filter, with a salinity of 32 practical salinity unit (PSU).

Three brands of artificial sea water were used, prepared to a salinity of 32 PSU:

Aquarium Systems Instant Ocean Sea Salt (from now referred to as Aquarium Systems) ( https://www.aquariumsystems.eu/sea-salt-instant-ocean-uk-c2x31922668)

TMC reef salt (from now referred to as TMC) ( https://www.tropicalmarinecentre.com/en/tmc/aquarium/water-chemistry/salt/reef-salt-10kg300l-bucket)

iQuatics Ocean Reef Pro Coral Salt (from now referred to as iQuatics) ( https://www.iquaticsonline.co.uk/product/iquatics-ocean-reef-pro-coral-aquarium-salt-20kg/)

### Chemical analysis of plastic leachates by Inductively Coupled Plasma – Optical Emission Spectrometry (ICP-OES)

Previous research has shown elevated zinc (Zn) concentrations, but not of other heavy metals, in PVC plastic pre-production nurdles using the same pellets as we use here ^10^. Zn concentrations were quantified using an Agilent 5110 VDV Inductively Coupled Plasma Optical Emission Spectrometer (ICP-OES) using the ICPExpert software version 7.6.2.12331 to control the instrument. Other selected elements were assessed to corroborate the absence of other heavy metals (see below). Analytical routines were followed as described in 10 with minimal modifications. All dilutions of chemical solutions used for analysis were done using 2 % v/v HNO _3_ prepared from concentrated Fisher Scientific General purpose grade nitric acid for which sufficient purity for ICP analysis was previously established. Dilution of the nitric acid was carried out using MilliQ water (18.2 MΩ) in a ratio of 1 part concentrated nitric acid in 32 parts MilliQ.

Zn concentrations were quantified using an acid blank and four calibration solutions made from a certified 1,000 µg/g Zn single element plasma standard (Fisher Scientific Specpure ^TM^). Calibrations were done before and after sample analysis to verify consistency of the calibration curves. Zn calibration solutions were prepared to nominal concentrations of 5.21 ng/g, 10.30 ng/g, 20.72 ng/g, and 52.22 ng/g using an intermediate stock solution of 1 µg/g that was prepared via another intermediate stock solution of 30 µg/g from the original certified standard. All stock solutions and standards were prepared gravimetrically to ensure optimal precision. Internal consistency of the measurements was ascertained using a synthetic Zn solution prepared to 25.94 ng/g which reproduced to 26.05 ng/g (n = 2) during the analytical sequence.

Potential biases resulting from seawater matrix were previously falsified using a suite of calibration solutions of the same standard in seawater matrix. Signals for all wavelengths were obtained in radial mode in six blocks of 30 seconds each, resulting in total signal integration of 180 s per sample. The peak intensity in counts per second was derived using the two nearest pixels to the expected peak centre with automatic baseline fitting applied. The Zn signal was quantified using the 213.857 nm and the Zn 202.548 nm lines.

In addition to quantitative Zn measurements, in additional analysis (presented in the Extended data, Chemical analysis 2) a series of metals were monitored and concentrations determined using a mixture of Inorganic Ventures multi element standards IV-ICPMS-71A and IV-ICPMS-71B resulting in nominal concentrations of 21.64 ng/g for Barium (Ba), Cadmium (Cd), Cobalt (Co), Chromium (Cr), Coper (Cu), Iron (Fe), Manganese (Mn), Nickel (Ni), Lead (Pb) and Vanadium (V), and 21.23 ng/g for Antimuonium (Sb) and Tin (Sn). Zn calibration solutions were prepared to nominal concentrations of 2.22 ng/g, 5.37 ng/g, 10.80 ng/g, and 21.29 ng/g using an intermediate stock solution of 1 µg/g that was prepared via another intermediate stock solution of 30 µg/g from the original certified standard. All stock solutions and standards were prepared gravimetrically to ensure optimal precision. Internal consistency of the measurements was ascertained using two synthetic Zn quality control solutions prepared to 4.31 ng/g and 8.58 ng/g which reproduced to 4.20 ng/g (n = 3) and 8.50 ng/g (n = 6) during the analytical sequence. Signals for all wavelengths were obtained in axial mode in five blocks of 40 seconds each, resulting in total signal integration of 200 s per sample. The peak intensity in counts per second was derived using the two nearest pixels to the expected peak centre with automatic baseline fitting applied. The Zn signal was quantified using the 213.857 nm line. The detection limit for Zn was determined as six times the standard deviation of the concentration estimate for the blank solution which was nominally devoid of Zn and found to be 0.13 ng/g, equivalent to c. 30 ng/g in the unknown solutions, accounting for an average dilution factor of c. 200 for the unkowns. Detection limits for the other elements were calculated as six times the standard deviation of concentrations obtained for the Zn quality control solutions which are nominally devoid of these metals. Wavelengths used for this quantification were Ba 455.403 nm; Cd 214.439 nm and 226.502 nm; Co 228.615 nm, 230.786 nm and 238.892 nm; Cr 205.560 nm and 267.716 nm; Cu 327.395 nm; Fe 259.940 nm; Mn 257.610 and 259.372 nm; Ni 231.604 nm and 231.604 nm; Pb 220.353 nm; Sb 206.834 nm and 217.582 nm; Sn 189.925 nm; V 292.401 nm. Where multiple wavelengths were used for quantification, the concentration estimates of individual analyses were pooled before averaging to improve detection limits. Resulting detection limits accounting for the average dilution factor of c. 200 for the unknown solutions convert to Ba = 4 ng/g; Cd = 20 ng/g; Co = 30 ng/g; Cr = 40 ng/g; Cu = 30 ng/g; Fe = 120 ng/g; Mn = 2 ng/g; Ni = 70 ng/g; Pb = 340 ng/g; Sb = 370 ng/g; Sn = 390 ng/g; V = 150 ng/g.

For all analysis, intensities of Calcium (Ca) (317.933 nm, 422.673 nm), Magnesium (Mg) (279.553 nm, 280.270 nm, 285.213 nm), Sodium (Na) (589.592 nm), Strontium (Sr) (407.771 nm, 421.552 nm), Sulphur (S) (181.972 nm) and Ba (455.403 nm) were monitored to verify the consistency of the sample dilution.

Plastic leachates were gravimetrically diluted c. 1:200 using 50 mL tubes in 2% nitric acid in order to adjust the sample matrix for optimal analytical conditions. Three independent leachate events for each type of water were analysed.

### *Ciona intestinalis* treatments

*C. intestinalis* were collected from Mylor Yacht Harbour (UK) in October 2023, transported to the lab within 30 minutes of collection and kept in clean natural seawater with aeration at 18 °C, with constant light to inhibit spawning. After a week, gametes were retrieved from both male and female gonads sequentially to avoid self-fertilisation. Crosses were done using single matings from male and female gonads from different animals. Eggs from each gonad were placed in 15 ml of FSW and dry sperm was diluted 15 µl to 10 ml of FSW, and 100 microliters of this dilution were used to fertilise the eggs. Fertilised eggs were left in a rotating platform for 10 minutes, after which they were washed once in FSW to prevent polyspermy. Washed zygotes were immediately transferred to the treatment solutions at a rate of 20 embryos per ml. Treatment solutions were each of the seawaters used, and PVC leachates in each type of seawater at a concentration of either 1% or 5% (v/v). Experiments were performed in triplicate using embryos from different parents for each replica. Images of 22 hours post fertilisation larvae were obtained using a Leica M165 C scope and a Leica DFC 295 camera.

### Image processing

Images in Figure 2 were individually adjusted for color balance, tone and exposure to obtain similar background colors using the levels tool in Photoshop CS 5.1. The current version of this program can be found at https://www.adobe.com/products/photoshop.html. A free alternative of this software can be used such as Fiji, which can be found at https://imagej.net/software/fiji/.

## Results and discussion

### Zn content in PVC plastic leachates

**Figure 1.**
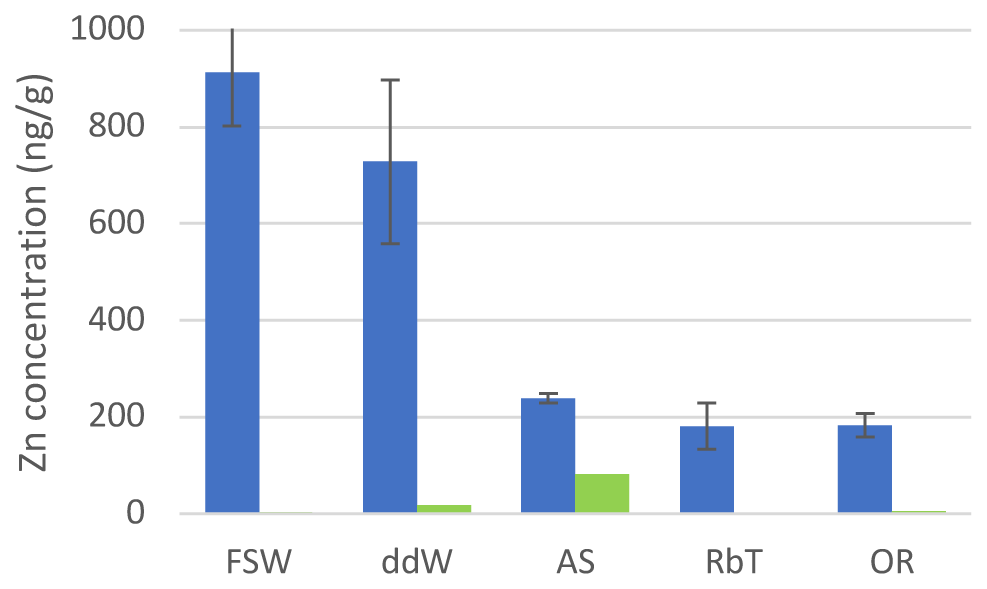
Zn concentrations in water. Concentration of Zn in 10% pre-production PVC nurdle leachates obtained in different natural and artificial waters used to treat *C. intestinalis* embryos (blue) and in their clean (with no pellets added) counterparts (green). FSW: filtered sea water; ddW: double distilled water; AS: Aquatic Systems Instant Ocean artificial sea water; RbT: TMC reef salt artificial seawater; OR: iQuatics Ocean Reef artificial sea water. See Methods for full artificial sea water information.

We monitored the zinc concentrations in 10% PVC nurdle leachates in normal sea water, double distilled water, and the three commercial brands of artificial sea water ( Figure 1; Extended data, Chemical Analysis 1 and 2) using inductively coupled plasma – optical emission spectrometry. Other metals were assessed quantitatively (Extended data, Chemical Analysis 2). We found that all three artificial sea waters used contained significantly less zinc originating from the plastics than filtered sea water or double distilled water.

**Figure 2.**
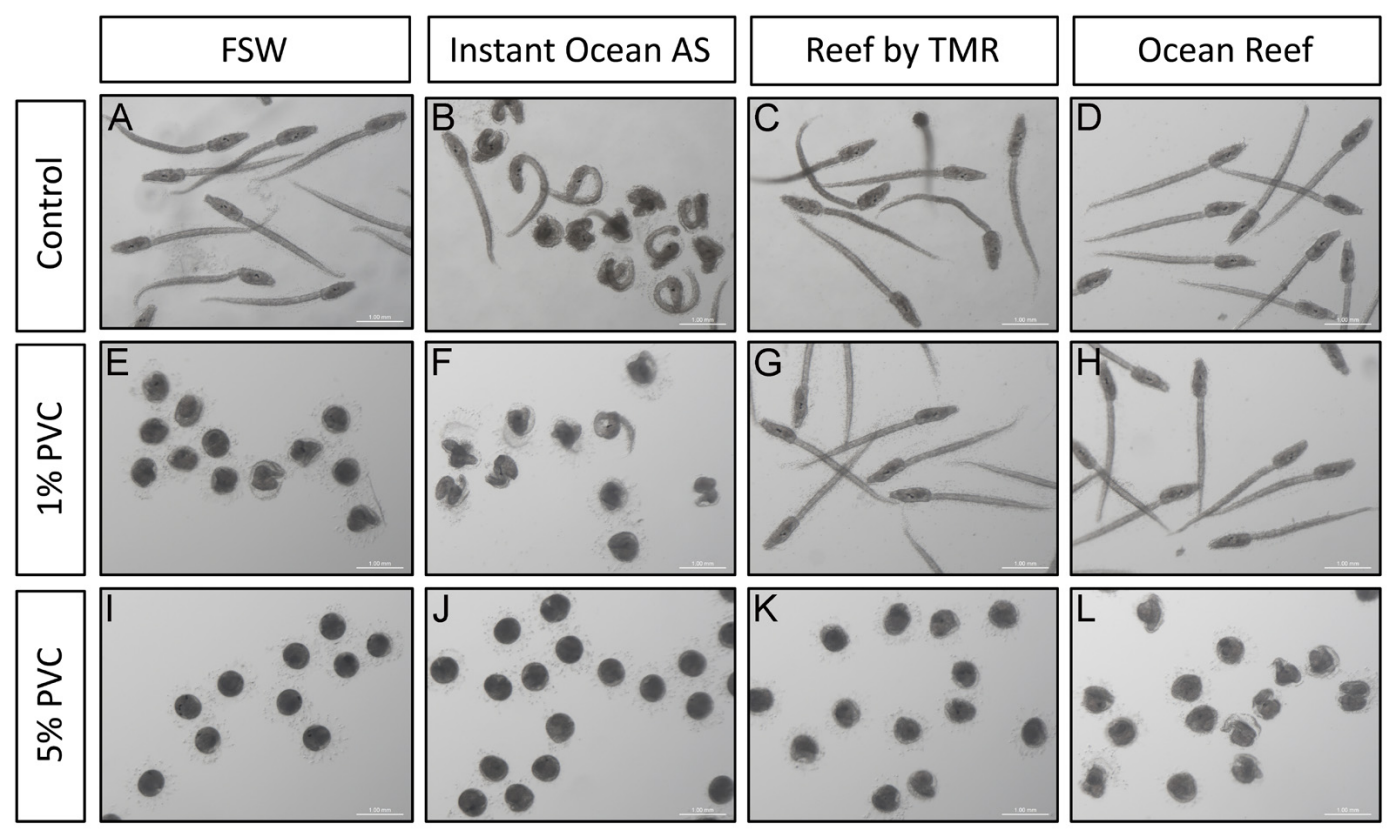
Effect on *C. intestinalis* development. 22 hours post fertilisation larvae of *C. intestinalis* grown in four types of clean seawater ( **A**– **D**) and in 1% ( **E**– **H**) and 5% ( **I**– **L**) PVC leachates obtained adding PVC pellets to those waters. Scale bar is 1 mm. All animals depicted come from the same batch. Individual panels have been level balanced to unify background colors.

We have previously shown high amounts of Zn leaching from PVC plastic pre-production nurdles into filtered sea water ^10^. Here, we repeat those experiments to find similar concentrations to the ones reported in that work (Paganos *et al*., 2023: n = 4, range = 0.78 to 1.18 μg/g, this work: n = 3, range 0.81 to 1.03 µg/g). These Zn concentrations are in average about one thousand times higher than that found naturally in seawater ^22^. However, the concentration of Zn due to PVC leachates found in the water is at least five times less when using any of the ASWs ( Figure 1, Extended data, Chemical analysis 1). Of the metals other than Zn that were monitored, the majority (Cd, Co, Cr, Ni, Pb, Sb, Sn, V) was found to be consistently below detection limit in all tested solutions (Extended data, Chemical analysis 2). A few tested solutions showed elevated levels of Cu (n=2; 50 ng/g, 392 ng/g) and Fe (n = 3; 270 ng/g; 330 ng/g; 2,400 ng/g) which is attributed to contamination, as these values are not consistent between solutions previous and after the leaching experiment. An increase of Ba linked to Zn increase has previously been related to the use of Zn-Ba mixed metals as liquid stabilisers. Indeed, the amount of Ba in PVC leachates increases as well in all water samples, except in Aquarium Systems ASW where the baseline Ba content is unusually high. Differences between pre- and post-leaching concentrations are statistically significant at the 95 % confidence level for all treatments apart from Aquarium Systems ASW, indicating that Ba is leached from plastic pellets during the experiment (Extended data, Chemical analysis 2). Water composition for the elements analysed is similar in all seawaters, expect for Mn. where levels above detection limit (2 ng/g) were observed in the majority of solutions but not in samples of de-ionised water. These correlate strongly (r ^2^ = 0.99) between pre- and post-leaching concentrations with a relationship of Mn _after leaching_ = 0.995 * Mn _before leaching_. This indicates that Mn was introduced via the salt rather than leached from plastic pellets during the experiment (Extended data, Chemical analysis 2). We do not relate this increase to the differences in Zn contents in our different seawater samples. Interestingly, Aquarium Systems ASW has an increased baseline of Zn of at least ten times higher than any of the other sea waters ( Figure 1 (green bars)).

### Leachates from PVC pre-production nurdles in FSW and ASW cause different intensity of developmental abnormalities in the tunicate *C. intestinalis*

We have previously shown that embryos from several species of marine invertebrates fail to grow properly when left to develop in these pre-production PVC pellet leachates ^21^. The tunicate *Ciona robusta* is particularly sensitive and displays aberrant phenotypes at lower concentrations of plastics than other animals. Here, we use the sister species *Ciona intestinalis* to see the effect the leachates in different waters have on embryo development.

*C. intestinalis* larvae exhibit two primary structures: the trunk, housing the adhesive organ, brain vesicle with pigmented sensory organs (otolith and ocellus), endoderm, and mesenchyme; and a straight tail for locomotion, containing the neural tube, notochord, endodermal strand, and muscles, all enveloped by the larval tunic. All control water treatments except Aquarium Systems ASW displayed a typical trunk and straight tail and vacuolated notochord cells ( Figure 2. A, C, D). However, larvae grown in Aquarium Systems ASW displayed strong abnormalities ( Figure 2. B): they developed shorter, coiled tails, and abnormal trunks, with misshaped adhesive organs and deformed sensory vesicles. We believe these abnormalities could be due to the high concentration of Zn found in this water (more than 80 ng/g; Figure 1, green bars). Other authors have found similar phenotypes when treating *C. intestinalis* with zinc chloride ^23^. Indeed, 1% PVC leachates in FSW displayed aberrations equivalent, if more intense, to the ones described for Aquarium Systems water before ( Figure 2. E): the tails were totally coiled, and most larvae had not managed to hatch from the chorions. Despite this, malformed trunks displayed sensory organs, demonstrating that morphogenesis had, to a certain extent, proceeded. These phenotypes were equivalent of those seen before in PVC leachate treated *C. robusta* larvae ^21^. 1% PVC leachate treatments in TMC or iQuatics waters did not give rise to any aberrations in the larvae ( Figure 2. G, H), showing that the concentration of Zn in these leachates (of around 18 ng/g) is not enough to create aberrations. Once the concentration of PVC particles increased to 5%, larvae failed to form in all treatments ( Figure 2. I-L), resulting in unhatched round individuals with pigmented spots, hinting at otolith and ocellus structures. Despite the strong malformations, larvae grown in 5% PVC leachates in TMC or iQuatics ASW displayed marginally less aberrant structures ( Figure 2. K, L).

The aberrations observed in this context follow a pattern of increased malformations with increased concentration of Zn in the water ( Figure 1, Figure 2). Hence, the malformations clearly correlate with the amount of Zn present in the leachates. We show that, if artificial sea water is used to obtain the leachates, the phenotypes observed can be less severe than in FSW, putatively leading to false negative results. Moreover, in highly sensitive animals like *C. intestinalis*, variations in the artificial sea water composition, like the one observed for Aquarium Systems ASW which contains increased amounts of Zn, can cause developmental problems that are not due to the treatments but to the experimental setup, potentially leading also to false positive results.

### Consequences for plastic contamination research

Extensive efforts are being made to catalogue the consequences of plastic contamination in different animals species, at different life cycle points (adults ^12, 24– 26^, embryos ^6, 7, 9, 10, 14^), using different toxicity routes (ingestion ^27– 30^, leachates ^6, 9, 12, 14, 17, 31– 33^, contact ^34, 35^) and physiological states (immunology ^12, 24, 36– 39^, reproduction ^12, 25, 40^, embryology ^6, 9, 10, 13, 14, 17, 32^ or behaviour ^41– 43^). A substantial part of this research is laboratory based, and scientists have so far been using indistinctively natural sea water and artificial sea water (formalised in Almeda *et al*., 2023 ^18^). Our results here show that research groups studying plastic toxicology may need to carry out additional tests to ensure that the water they are using reflects either environmental conditions or, at least, does not hinder their results and the conclusions they reach. If utilising artificial sea water can change the concentration of Zn retrieved from plastic preproduction pellets, like we demonstrate here, it may have other behaviours that may also change the dynamics of action of plastic leachates for other chemicals, including molecules known to leach from plastics such as Polycyclic aromatic hydrocarbons, phthalates, polychlorinated biphenyls and others ^5^. In the case of plastic leachates, if what researchers intend is to mimic environmental conditions, it would be important to ascertain the chemical composition of such waters, and if this is artificial sea water, to be able to contrast it with the composition of leachates obtained in natural sea water. We cannot state the reach of our findings for other types of experiments, for instance when the studied interaction is using other types of plastic particles or nanobeads, but our results would suggest that comparisons between ASW and FSW leachates should be performed when using these other particles in ASW.

## Conclusions

Artificial and natural sea water have different behaviours when leaching heavy metals from pre-production PVC particles. This may be extendable to other chemicals leaching from these particles, and also to other particles or types of plastic material. The leachates obtained in artificial and natural seawater using these plastics do not have the same effects on the development of the tunicate *C. intestinalis*. Moreover, some artificial water compositions may not be ideal for embryo-toxicology tests and may lead to increased aberrations. Our findings suggest that artificial and natural sea water types are not comparable, emphasizing the need for caution or additional tests when drawing conclusions from data obtained in artificial seawater.

## Ethics and consent

*C. intestinalis* were collected from Mylor Yacht Harbour (UK) with permission of the company. *C. intestinalis* is not covered under EU Directive 2010/63/EU of the European Parliament and the Council of 22 September 2010 on the protection of animals used for scientific purposes nor in the Animals Scientific Procedures Act (ASPA) (UK). Animal handling was in accordance with the guidelines of our academic institutions. The least number of animals were used to perform the experiments while still permitting to have sufficient replicas to validate the results.

## Data Availability

Figshare: "Extended data for Natural sea water and artificial sea water are not equivalent in plastic leachate contamination studies", https://doi.org/10.6084/m9.figshare.25127069 ^44^. This project contains the following underlying data: Chemical analysis 1 spreadsheet data Raw, unedited, uncropped images Rebuttal chemical analysis of leachates (Chemical analysis 2) Data are available under the terms of the Creative Commons Attribution 4.0 International license (CC-BY 4.0).
