# Supplementary material for: Natural sea water and artificial sea water are not equivalent in plastic leachate contamination studies
Source: Open Res Eur. 2024 Oct 17;4:59. Originally published 2024 Mar 21. [Version 3] doi: 10.12688/openreseurope.17112.3 (PMC11427872; doi:10.12688/openreseurope.17112.3)
Supplement: Peer review history

# Reviewer report: Reviewer response for version 3

**DOI:** 10.21956/openreseurope.20145.r45221

**Published:** 2024-10-30

**Authors:** Atsuko Sato

**Affiliations:** 1 Department of Biology, Ochanomizu University, Tokyo, Japan

My points are now clarified and I am happy to their revised version.

Is the study design appropriate and does the work have academic merit?

Yes

Is the work clearly and accurately presented and does it cite the current literature?

Yes

If applicable, is the statistical analysis and its interpretation appropriate?

Not applicable

Are all the source data underlying the results available to ensure full reproducibility?

Yes

Are the conclusions drawn adequately supported by the results?

Yes

Are sufficient details of methods and analysis provided to allow replication by others?

Yes

Reviewer Expertise:

NA

I confirm that I have read this submission and believe that I have an appropriate level of expertise to confirm that it is of an acceptable scientific standard.

# Reviewer report: Reviewer response for version 2

**DOI:** 10.21956/openreseurope.19843.r43728

**Published:** 2024-09-26

**Authors:** Seren Acarer Arat

**Affiliations:** 1 Environmental Engineering Department, Istanbul University-Cerrahpaşa, Avcılar, Turkey

The authors have sufficiently fulfilled the issues specified in the revision stage. The author must ensure that all abbreviations (PVC, Zn, etc.) in the article are defined. Abbreviations should be defined where they first appear. 

 Since the number of studies examining the additives released from plastics into the aquatic environment and their effects on living organisms is limited, I think this study will contribute to the literature. I think this study can be accepted after a major revision.

 Introduction

 “Plastics also can contain chemical additives which are added to the polymers during the manufacturing process or that can be adsorbed and accumulated from the environment.” The subject of the article is related to substances leaching from plastics.  Therefore, immediately after this sentence, it should be comprehensively mentioned which substances can leach from plastics into the aquatic environment with references.

 Methods

 Information on the physical and/or chemical properties of the PVC used in the leaching studies or a detailed table can be added.

 Results and discussion

 I strongly recommend FTIR analysis of the PVC used in this study and evaluation of the FTIR spectrum.

 I would also like to see the amount of microplastics released from PVC in contact with different types of water in this study.

Is the study design appropriate and does the work have academic merit?

Yes

Is the work clearly and accurately presented and does it cite the current literature?

Yes

If applicable, is the statistical analysis and its interpretation appropriate?

Yes

Are all the source data underlying the results available to ensure full reproducibility?

Yes

Are the conclusions drawn adequately supported by the results?

Yes

Are sufficient details of methods and analysis provided to allow replication by others?

Yes

Reviewer Expertise:

microplastics

I confirm that I have read this submission and believe that I have an appropriate level of expertise to confirm that it is of an acceptable scientific standard, however I have significant reservations, as outlined above.

# Response

**Published:** 2024-10-08

**Authors:** Eva Jimenez Guri

**Affiliations:** BEOM, Stazione Zoologica Anton Dohrn Napoli, Naples, Campania, Italy

We thank the reviewer for their positive comments. Below we give our replies to their concerns.

**1. The author must ensure that all abbreviations (PVC, Zn, etc.) in the article are defined. Abbreviations should be defined where they first appear.**

 Ans: We have modified the text to define all abbreviations as suggested by the reviewer.

**2. “Plastics also can contain chemical additives which are added to the polymers during the manufacturing process or that can be adsorbed and accumulated from the environment.” The subject of the article is related to substances leaching from plastics.  Therefore, immediately after this sentence, it should be comprehensively mentioned which substances can leach from plastics into the aquatic environment with references.**

**Ans: **We have mentioned the substances that can leach from the plastics and references are provided. It now reads:

*Plastics also can contain chemical additives which are added to the polymers during the manufacturing process to enhance desired characteristics, such as plasticizers, biocides, flame retardants, foaming agents, pigments, stabilisers, and many more (summarised in a comprehensive review in [ https://doi.org/10.1016/j.watres.2020.116170]), or that can be adsorbed and accumulated from the environment ^3, 4^ .*

**3. Information on the physical and/or chemical properties of the PVC used in the leaching studies or a detailed table can be added.**

 Ans: We have added information on the physical characteristics of the PVC pellets used. It reads:

*These were flexible tubular transparent pellets with a slight blue hue and average dimensions of 3.2 × 3.7 mm.*

**4. I strongly recommend FTIR analysis of the PVC used in this study and evaluation of the FTIR spectrum.   I would also like to see the amount of microplastics released from PVC in contact with different types of water in this study.**

 Ans: We agree with the reviewer that both things would be interesting points to look at in the future, specially checking the waters for the amounts of microplastics released. While we are not able to do this for the present manuscript, we will factor this in in our further research. Notwithstanding, we believe in the relevance of our results without the experiments suggested by the reviewer, as we can demonstrate that the amounts of zinc leached from the PVC nurdles change depending on which type of seawater, artificial or natural, one is using, proving the point of the research that not all waters are equivalent when used to obtain leachates from plastics.

# Reviewer report: Reviewer response for version 2

**DOI:** 10.21956/openreseurope.19843.r42797

**Published:** 2024-09-11

**Authors:** Atsuko Sato

**Affiliations:** 1 Department of Biology, Ochanomizu University, Tokyo, Japan

The author's replies are all good and my previous suggestions and comments are all responded. 

 However one thing I still do not understand is why, in Fig 2, B looks very different from A, C and D. According to the author, embryos are collected from the same adults and A-D should look similar as control experiments. 

 If we get a convincing answer to the difference or better pictures from two other replicates, I will  approve this manuscript.

Is the study design appropriate and does the work have academic merit?

Yes

Is the work clearly and accurately presented and does it cite the current literature?

Yes

If applicable, is the statistical analysis and its interpretation appropriate?

Not applicable

Are all the source data underlying the results available to ensure full reproducibility?

Yes

Are the conclusions drawn adequately supported by the results?

Yes

Are sufficient details of methods and analysis provided to allow replication by others?

Yes

Reviewer Expertise:

NA

I confirm that I have read this submission and believe that I have an appropriate level of expertise to confirm that it is of an acceptable scientific standard, however I have significant reservations, as outlined above.

# Response

**Published:** 2024-09-11

**Authors:** Eva Jimenez Guri

**Affiliations:** BEOM, Stazione Zoologica Anton Dohrn Napoli, Naples, Campania, Italy

We thank the reviewer for their positive comments, and would like to clarify their doubts about Figure 2B. Indeed, all larvae in the Figure 2 are from the same fertilisation event. The fact that panel B (control for AS water) does not look like the other controls is because we have found that AS salt has an increase in Zn content as compared with other artificial seawaters and filtered seawater. Because of this, we believe the malformations seen in the AS seawater when no leachates are used is because of this higher concentration of Zn. This is stated in the results when explaining Figure 2 and the phenotypes observed in *C. Intestinalis* larvae:

- However, larvae grown in AS ASW displayed strong abnormalities (Figure 2. B): they developed shorter, coiled tails, and abnormal trunks, with misshaped adhesive organs and deformed sensory vesicles. We believe these abnormalities could be due to the high concentration of Zn found in this water (more than 80 ng/g; Figure 1, green bars).

At the end of this section we also comment on this observation with this discussion:

- Moreover, in highly sensitive animals like *C. intestinalis*, variations in the artificial sea water composition, like the one observed for AS which contains increased amounts of Zn, can cause developmental problems that are not due to the treatments but to the experimental setup, potentially leading also to false positive results.

And in the conclusions we refer to the fact that some artificial seawaters (like AS in the case of this report) may not be suitable for developmental experiments as their salt composition may lead to aberrations that are not linked to the experiment but to the salt used:

- Moreover, some artificial water compositions may not be ideal for embryotoxicology tests and may lead to increased aberrations.

We hope that this clarifies the reviewer’s doubts.

# Reviewer report: Reviewer response for version 2

**DOI:** 10.21956/openreseurope.19843.r42798

**Published:** 2024-08-06

**Authors:** Amitava Mukherjee

**Affiliations:** 1 Centre for Nanobiotechnology, Vellore Institute of Technology, Vellore, Tamil Nadu, India

My concerns with the previous submission are well addressed by the authors in the revised version. The revised version is acceptable.

Is the study design appropriate and does the work have academic merit?

Partly

Is the work clearly and accurately presented and does it cite the current literature?

Partly

If applicable, is the statistical analysis and its interpretation appropriate?

Yes

Are all the source data underlying the results available to ensure full reproducibility?

Partly

Are the conclusions drawn adequately supported by the results?

Partly

Are sufficient details of methods and analysis provided to allow replication by others?

Partly

Reviewer Expertise:

Nano ecotoxicology; Toxicloogy of Micro nano plastics;

I confirm that I have read this submission and believe that I have an appropriate level of expertise to confirm that it is of an acceptable scientific standard.

# Reviewer report: Reviewer response for version 1

**DOI:** 10.21956/openreseurope.18495.r41337

**Published:** 2024-06-17

**Authors:** Amitava Mukherjee

**Affiliations:** 1 Centre for Nanobiotechnology, Vellore Institute of Technology, Vellore, Tamil Nadu, India

The problem statement and hypothesis:

 Whether the authors exclude the possibility of leaching any organic additives in the current study design and hypothesis should be spelled out. Whether the medium have any role in releasing the organic additives too? If they restrict the study to the effects of heavy metal alone, this should be clearly stated in the title, abstract, and introduction too. “Leachate’ term may be used in a wider context and this may be misleading to the readers.

 Environmental relevance:

 The environmental relevance of the work, and how the differences between three different seawater media would reflect real environmental scenarios and eco-toxicological context can be briefly added. They added a section later in the report and a few lines can be added in the introduction and abstract on this point too.

 Methods:

 The authors should add briefly the leaching protocol in this study unless the reader is forced to go back to their previous publications.

 Results and discussion:

 Why did the authors select Zn alone in the results principally? As per their previous study, Zn predominantly came from the PVC, whether changing the ASW mediums had any impact on the leachability of other inorganics.  Please comment on this aspect.

 In the case of toxicity tests with *Ciona robusta, *whether the authors have used a Zn salt control (standard addition to the medium) with equivalent concentrations to drive home the point.

Is the study design appropriate and does the work have academic merit?

Partly

Is the work clearly and accurately presented and does it cite the current literature?

Partly

If applicable, is the statistical analysis and its interpretation appropriate?

Yes

Are all the source data underlying the results available to ensure full reproducibility?

Partly

Are the conclusions drawn adequately supported by the results?

Partly

Are sufficient details of methods and analysis provided to allow replication by others?

Partly

Reviewer Expertise:

Nano ecotoxicology; Toxicloogy of Micro nano plastics;

I confirm that I have read this submission and believe that I have an appropriate level of expertise to confirm that it is of an acceptable scientific standard, however I have significant reservations, as outlined above.

# Response

**Published:** 2024-07-28

**Authors:** Eva Jimenez Guri

**Affiliations:** BEOM, Stazione Zoologica Anton Dohrn Napoli, Naples, Campania, Italy

**Reviewer 2 Comment:**

**The problem statement and hypothesis:**

**Whether the authors exclude the possibility of leaching any organic additives in the current study design and hypothesis should be spelled out. Whether the medium have any role in releasing the organic additives too? If they restrict the study to the effects of heavy metal alone, this should be clearly stated in the title, abstract, and introduction too. “Leachate’ term may be used in a wider context and this may be misleading to the readers.**

 Author Response: The reviewer is right in this comment. We have focused on the leaching of heavy metals since our previous experiments clearly showed that Zn (and no other heavy metals) were strongly released from these particles onto filtered sea water. Checking for organic and other compounds was not available to us at the moment of this research, but finding this difference on the Zn content already was telling us that there was a difference in the leaching of compounds between ASW and FSW.  We have stressed that we focused our experiment on the leachates of heavy metals in the abstract and introduction and emphasised that we have not ruled out the possibility of other molecules, including organic additives, can be leached from the plastics, despite not having measured them in this case.  

**Reviewer 2 Comment:**

**Environmental relevance:**

**The environmental relevance of the work, and how the differences between three different seawater media would reflect real environmental scenarios and eco-toxicological context can be briefly added. They added a section later in the report and a few lines can be added in the introduction and abstract on this point too.**  

 Author Response: We do not believe that the different artificial sea waters could reflect natural environmental scenarios. We wanted to report that artificial sea water in experimental settings working on plastic leachates may not be always reflecting what natural sea water is doing. We have clarified this in the abstract and the introduction as suggested by the reviewer.  

**Reviewer 2 Comment:**

**Methods:**

**The authors should add briefly the leaching protocol in this study unless the reader is forced to go back to their previous publications.**

 Author Response: We have extended the explanation for the leaching protocol.  

**Reviewer 2 Comment:**

**Results and discussion:**

**Why did the authors select Zn alone in the results principally? As per their previous study, Zn predominantly came from the PVC, whether changing the ASW mediums had any impact on the leachability of other inorganics.  Please comment on this aspect.**

 Author Response: We were conducting experiments on animals reared in ASW and on animals reared in FSW. Only on those animals where the leachates were done in ASW were not presenting clear disruptive phenotypes, whereas when using FSW leachates we did see aberrant phenotypes. We then used different ASW, and the results were different again, where both controls and treated had problems. We then decided to check if this could be due to differences between in the ASW and the FSW, and between different ASWs. We looked at the presence of other heavy metals and other elements in the PVC leachates (Ba, Cd, Co, Cr, Cu, Fe, Mn, Ni, Pb, Sb, Sn, V, Zn) and did not find any significant difference in the composition for any other than Zn (and Ba and Mn as discussed in the manuscript). However, we did not record all those measurements. We have now looked at the presence of those elements in the different waters analysed (preparing leachates of the PVC pellets in six new independent aliquots of water from each type of salt). We provide the measurements in a new table in the Extended Data, Chemical Analysis 2. We see the same trend as we discussed in the manuscript, except for trace amounts of coper in some of the samples, very close to the limit of detection of the instrument. Moreover, individual aliquots show a spike in some elements (only Fe and Co clearly over the limit of detection of the instrument) for a few samples. These spikes are not uncommon and are attributed to punctual contamination during the manipulation of the samples in the ICP-OES instrument, as these values are not consistent between solutions previous and after the leaching experiment. We have added this information in the manuscript, as well as providing the new analysis in the Extended data, Chemical analysis 2

**Reviewer 2 Comment:**

**In the case of toxicity tests with Ciona robusta, whether the authors have used a Zn salt control (standard addition to the medium) with equivalent concentrations to drive home the point.**

 Author Response: We have not conducted this experiment. However, there is other literature showing the effects of Zn on *Ciona* showing equivalent phenotypes (Gallo et al 2011, Marine Ecology). This reference is now added to the manuscript.

# Reviewer report: Reviewer response for version 1

**DOI:** 10.21956/openreseurope.18495.r39551

**Published:** 2024-04-28

**Authors:** Atsuko Sato

**Affiliations:** 1 Department of Biology, Ochanomizu University, Tokyo, Japan

This work is very important in the research field concerning the impact of plastics to the oceans. The results presented were unexpected, and should be made aware to other researchers in the field.

 This work presents the results in a qualitative as well as a quantitative way; however, the way of replicates should be clarified. Experiments on embryonic development were performed triplicate; whether this was done with the same batch (cross from eggs from the same mother and sperm from the same father) or different three batches, were not clear. Presumably there are individual differences in susceptibility. In Figure 2, it should also mentioned in the legend whether the pictures are from the same batch or not (it should be the same batch, because of the reason as above). Since the control phenotype in AS is apparently different from TMR or Reef, I was not sure whether we could compare the difference in the impact of leachates between AS and TMR or AS and OR. The large difference in the impact between AS and TMR or AS and OR might be due to difference in the individual batches.

 Also, to conclude whether difference in the concentration of Zn affected the results, differences in Zn concentration between AS, RbT and OR needs to be assessed statistically.

 Also I should point out that there is a very minor mistake: page 5 left column, 'crosses were done using single mattings' should probably be 'single matings'.

Is the study design appropriate and does the work have academic merit?

Yes

Is the work clearly and accurately presented and does it cite the current literature?

Yes

If applicable, is the statistical analysis and its interpretation appropriate?

Not applicable

Are all the source data underlying the results available to ensure full reproducibility?

Yes

Are the conclusions drawn adequately supported by the results?

Yes

Are sufficient details of methods and analysis provided to allow replication by others?

Yes

Reviewer Expertise:

NA

I confirm that I have read this submission and believe that I have an appropriate level of expertise to confirm that it is of an acceptable scientific standard, however I have significant reservations, as outlined above.

# Response

**Published:** 2024-07-28

**Authors:** Eva Jimenez Guri

**Affiliations:** BEOM, Stazione Zoologica Anton Dohrn Napoli, Naples, Campania, Italy

**Reviewer 1 Comment:** 

**This work is very important in the research field concerning the impact of plastics to the oceans. The results presented were unexpected, and should be made aware to other researchers in the field**.

 Author Response: We thank the reviewer for their positive comments about our article.

**Reviewer 1 Comment:** 

**This work presents the results in a qualitative as well as a quantitative way; however, the way of replicates should be clarified. Experiments on embryonic development were performed triplicate; whether this was done with the same batch (cross from eggs from the same mother and sperm from the same father) or different three batches, were not clear. Presumably there are individual differences in susceptibility.**

 Author Response: The triplicates were performed using different adult animals for each replica. This has been clarified in the methods section.  

**Reviewer 1 Comment:** 

**In Figure 2, it should also mentioned in the legend whether the pictures are from the same batch or not (it should be the same batch, because of the reason as above).**

 Author Response: The pictures are all from the same batch of animals. This is now stated in the figure legend.  

**Reviewer 1 Comment:** 

**Since the control phenotype in AS is apparently different from TMR or Reef, I was not sure whether we could compare the difference in the impact of leachates between AS and TMR or AS and OR. The large difference in the impact between AS and TMR or AS and OR might be due to difference in the individual batches.**

 Author Response: All exposures were done in triplicate with three different batches of eggs and sperm from different adults. The three batches showed the same phenotype, the same susceptibility of AS as compared to the other artificial sea waters or FSW. Therefore we are confident the differences we see are because of three different type of water rather than because of susceptibility of the individual batches.  

**Reviewer 1 Comment:** 

**Also, to conclude whether difference in the concentration of Zn affected the results, differences in Zn concentration between AS, RbT and OR needs to be assessed statistically.**

 Author Response: We have done the analysis of Zn concentrations in the artificial sea waters again, preparing six independent aliquots of water from each type of salt. The Zn concentrations were determined using the 213.857 nm line, as described in the Materials and Methods. The limit of detection was 13 ng/g, so anything below that limit was not quantified and values have been set to zero. The readings are provided in the table below, and the differences between the Zn concentration in the samples of each artificial sea water with no plastic added is significantly different between AS and RbT or OR with a p value of 0.003355 using an ANOVA test. Type of water Zn in AS (ng/g) Zn in RbT (ng/g) Zn in OR (ng/g) Aliquot 1 78 0 0 Aliquot 2 59 0 0 Aliquot 3 0 0 0 Aliquot 4 92 0 0 Aliquot 5 49 0 0 Aliquot 6 0 0 0   We do not want to alter Figure 1 to add these results, as those measurements were done in the waters used for the exposure experiments in the *C. intestinalis* embryos and the bars of the “clean” water correspond to the same water as was used for the preparation of the leachates. Also, note that two of the readings of AS were below the limit of detection. We believe this is because the Zn particles in the salt mix are punctual, so some aliquots of salt may stochastically contain more Zn than others.  

**Reviewer 1 Comment:**

**Also I should point out that there is a very minor mistake: page 5 left column, 'crosses were done using single mattings' should probably be 'single matings'.**

 Author Response: This was indeed a mistake and has now been corrected.
